# Supplementary figures and images for: Novel strains of Tomato Spotted Wilt Orthotospovirus (TSWV) are transmitted by western flower thrips in a context-specific manner
Source: PLoS One. 2025 Jul 10;20(7):e0323037. doi: 10.1371/journal.pone.0323037 (PMC12244803; doi:10.1371/journal.pone.0323037)

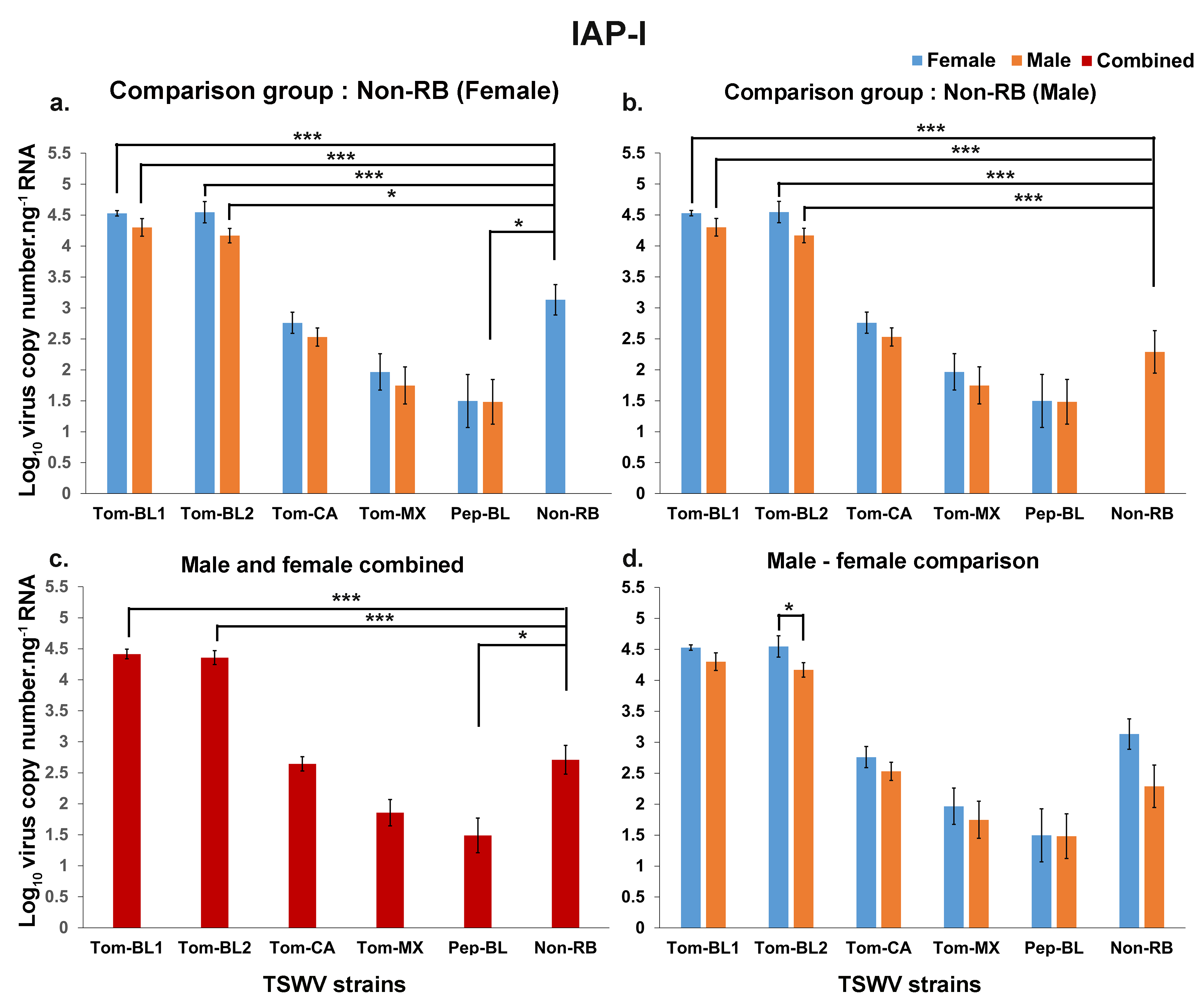

Supplement: S1 Fig — Average copy number of different TSWV strains transmitted by female (n = 10) and male (n = 10) thrips, and compared to Non-RB strain transmitted by either (a) female; or (b) male (c) average copy number of different strains inoculated by F. occidentalis (male and female combined) compared to Non-RB strain inoculated by F. occidentalis (male and female combined) (d) comparison of average copy number of different TSWV strains inoculated by female vs male within the strains. Asterisks indicate significant differences at α = 0.05 (*P < 0.05, **P < 0.01, ***P < 0.001). (TIF) [file pone.0323037.s001.tif]

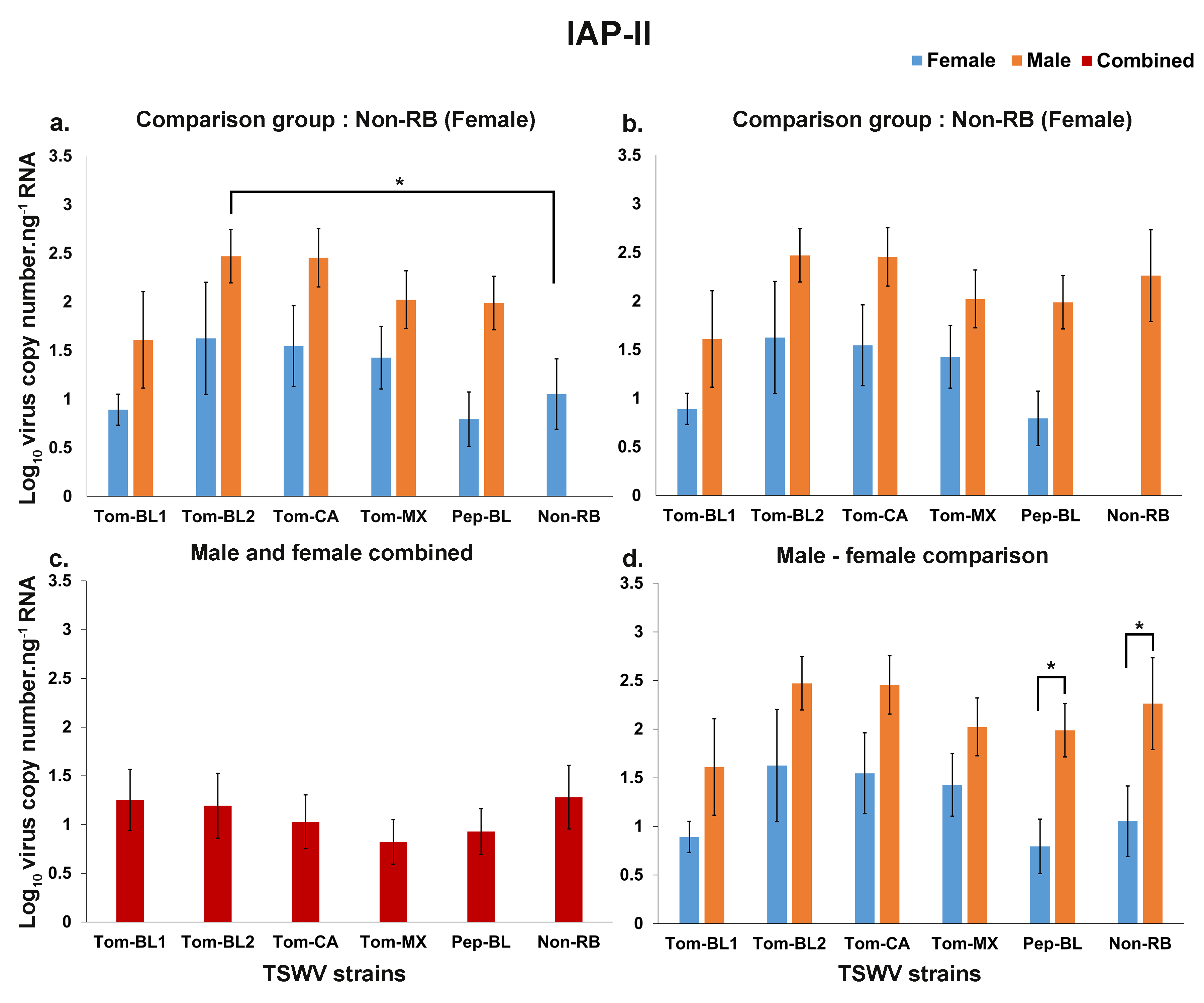

Supplement: S2 Fig — Average copy number of different TSWV strains transmitted by female (n = 10) and male (n = 10) thrips, and compared to Non-RB strain transmitted by either (a) female; or (b) male (c) average copy number of different strains inoculated by F. occidentalis (male and female combined) compared to Non-RB strain inoculated by F. occidentalis (male and female combined) (d) comparison of average copy number of different TSWV strains inoculated by female vs male within the strains. Asterisks indicate significant differences at α = 0.05 (* P < 0.05, ** P < 0.01, *** P < 0.001). (TIF) [file pone.0323037.s002.tif]

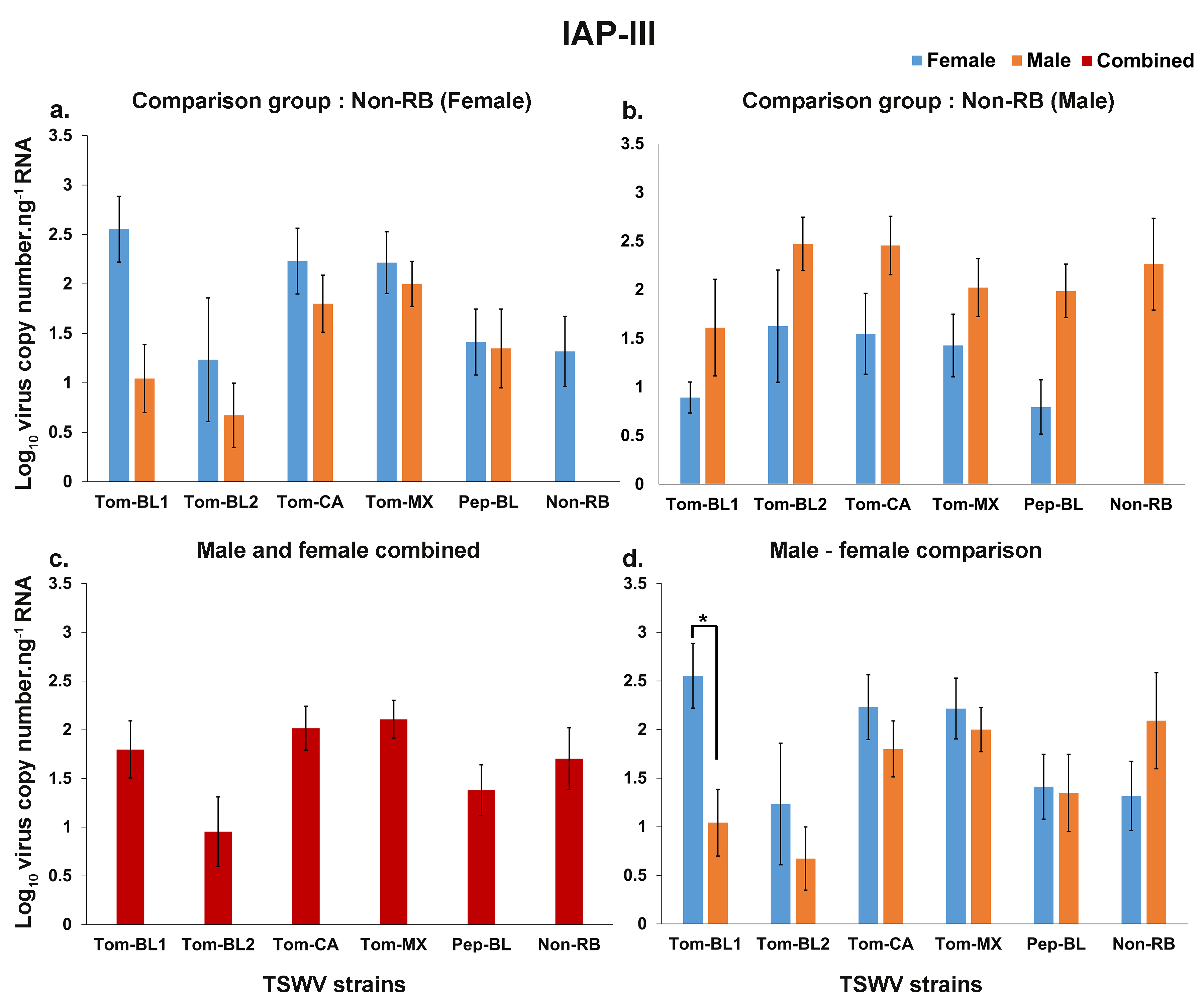

Supplement: S3 Fig — Average copy number of different TSWV strains transmitted by female (n = 10) and male (n = 10) thrips, and compared to Non-RB strain transmitted by either (a) female; or (b) male (c) average copy number of different strains inoculated by F. occidentalis (male and female combined) compared to Non-RB strain inoculated by F. occidentalis (male and female combined) (d) comparison of average copy number of different TSWV strains inoculated by female vs male within the strains. Asterisks indicate significant differences at α = 0.05 (*P < 0.05, **P < 0.01, ***P < 0.001). (TIF) [file pone.0323037.s003.tif]

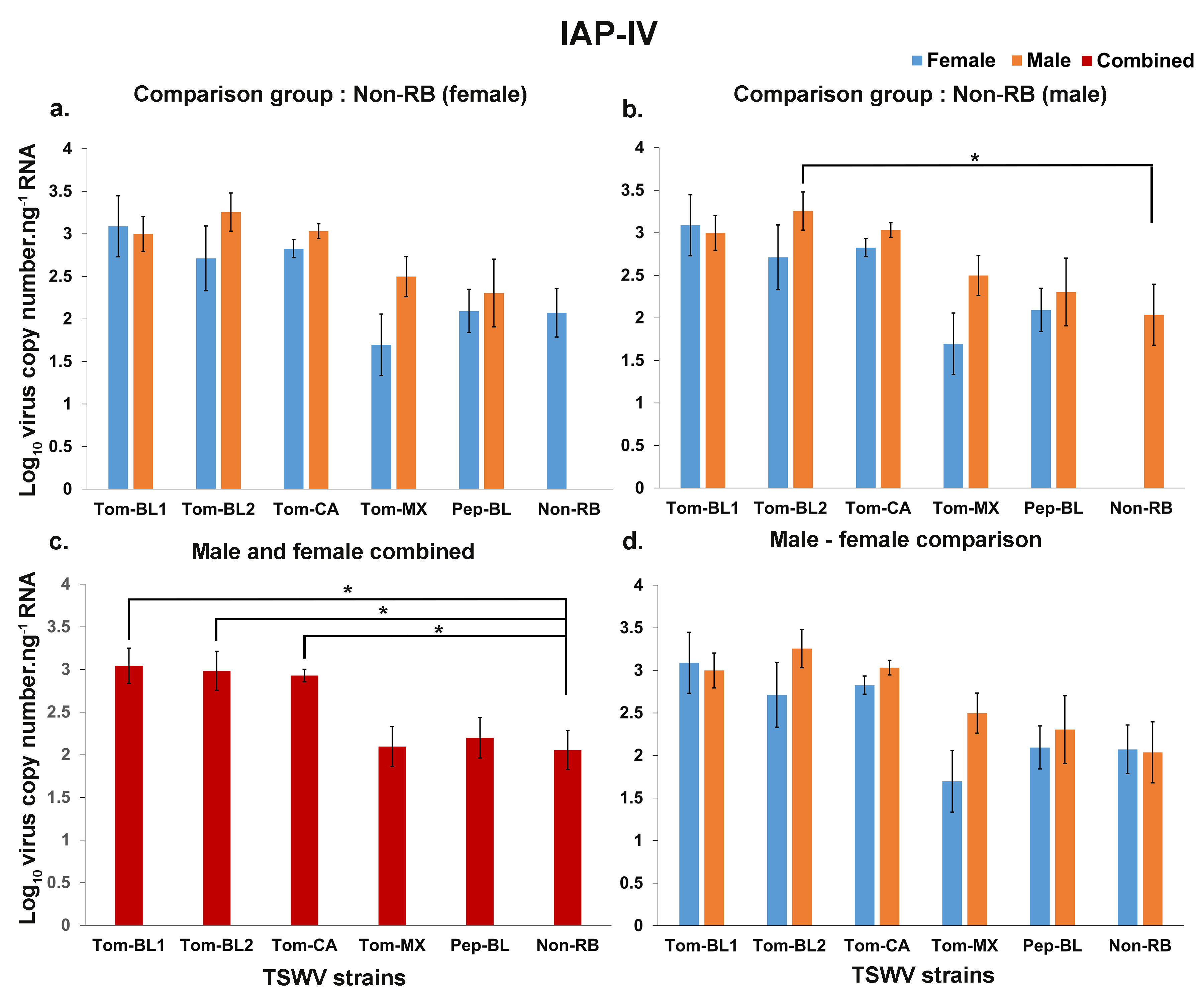

Supplement: S4 Fig — Average copy number of different TSWV strains transmitted by female (n = 10) and male (n = 10) thrips, and compared to Non-RB strain transmitted by either (a) female; or (b) male (c) average copy number of different strains inoculated by F. occidentalis (male and female combined) compared to Non-RB strain inoculated by F. occidentalis (male and female combined) (d) comparison of average copy number of different TSWV strains inoculated by female vs male within the strains. Asterisks indicate significant differences at α = 0.05 (*P < 0.05, **P < 0.01, ***P < 0.001). (TIF) [file pone.0323037.s004.tif]

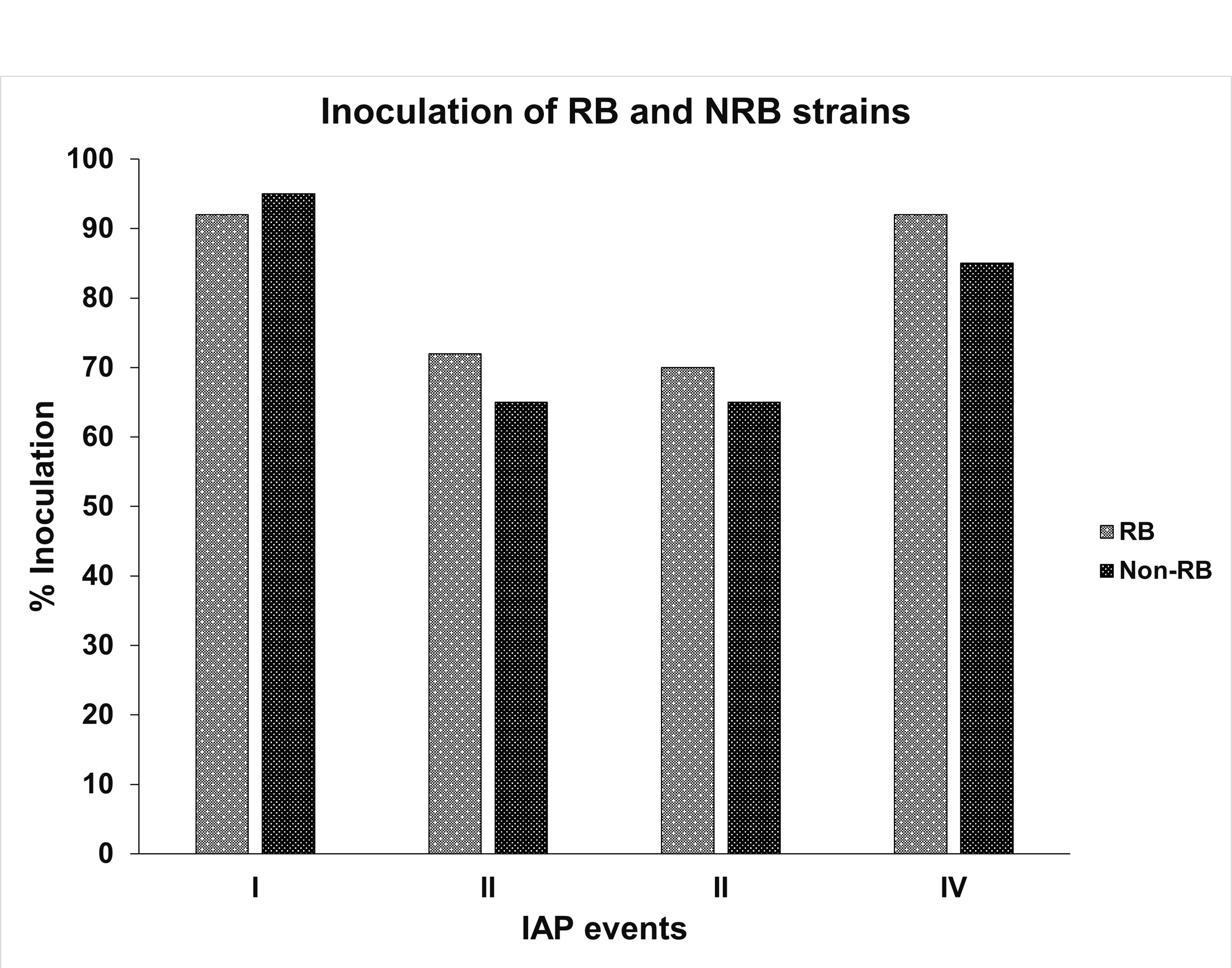

Supplement: S5 Fig — (TIF) [file pone.0323037.s005.tif]

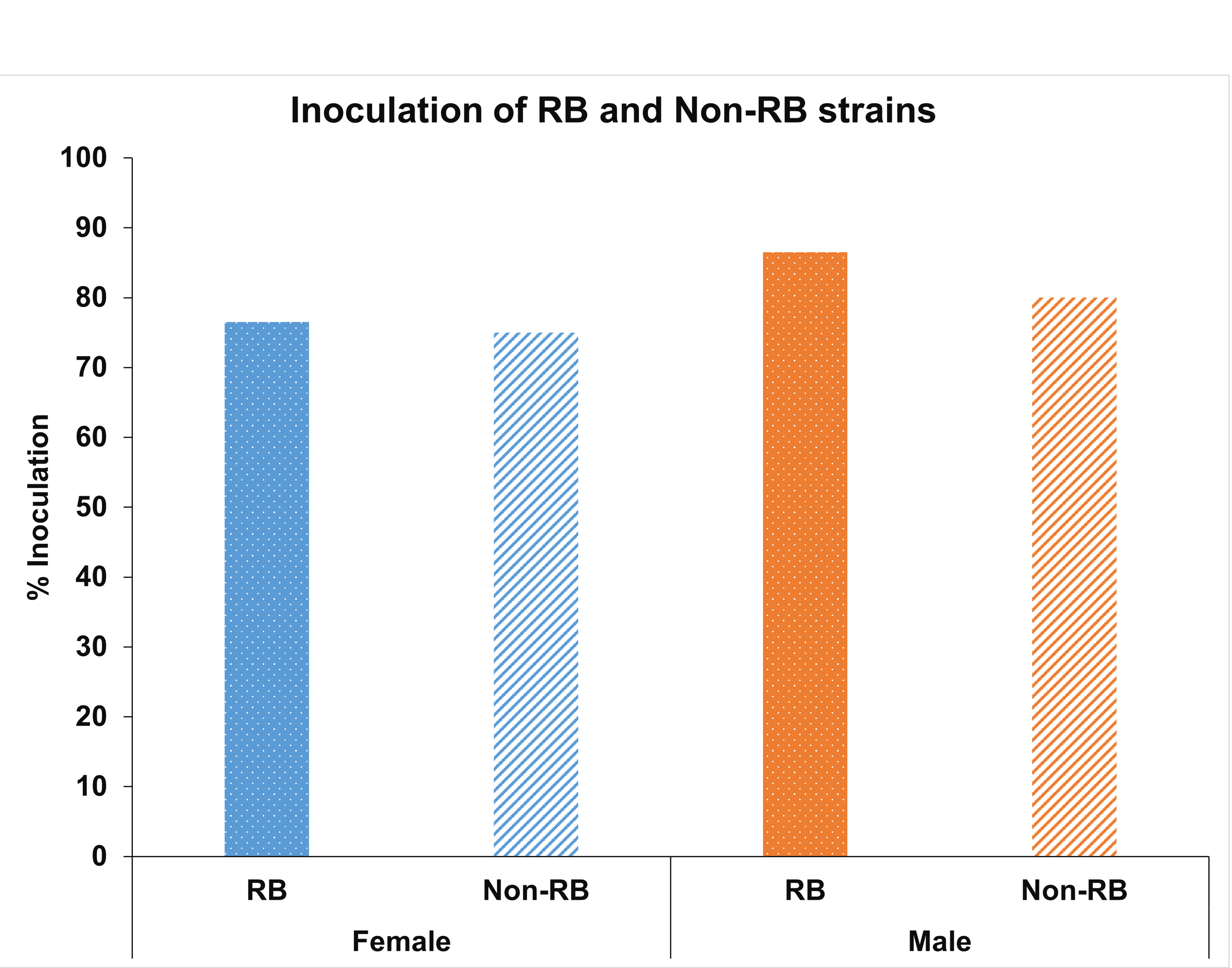

Supplement: S6 Fig — (TIF) [file pone.0323037.s006.tif]

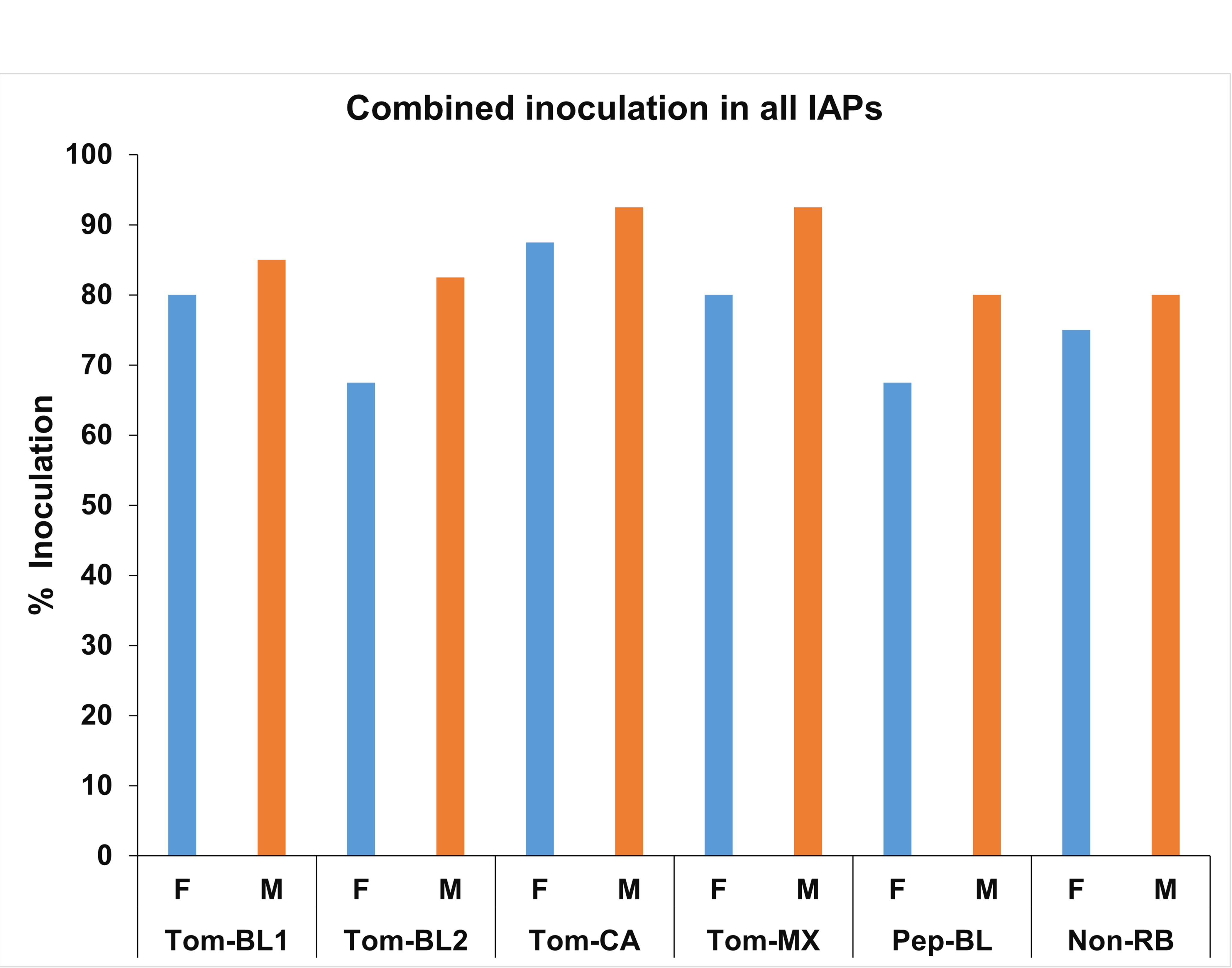

Supplement: S7 Fig — (TIF) [file pone.0323037.s007.tif]
