# Supplementary material for: Novel strains of Tomato Spotted Wilt Orthotospovirus (TSWV) are transmitted by western flower thrips in a context-specific manner
Source: PLoS One. 2025 Jul 10;20(7):e0323037. doi: 10.1371/journal.pone.0323037 (PMC12244803; doi:10.1371/journal.pone.0323037)
Supplement: Attachment — Submitted filename: Cover letter.pdf [file pone.0323037.s010.pdf]

Texas A&M AgriLife Research
Texas A&M University Entomology

Dr Sumit Jangra, Ph.D.
Academic Editor
PLOS One

June 17, 2025

Dear Dr Jangra,

Once again, we sincerely thank you and the reviewers for your thoughtful, constructive, and insightful feedback on our revised manuscript, "***Novel strains of tomato spotted wilt orthotospovirus (TSWV) are transmitted by western flower thrips in a context-specific manner***." While most of the comments were minor, they have nonetheless helped meaningfully improve the clarity and overall quality of the manuscript. We have carefully addressed all concerns raised, including clarifying the data presentation and the rationale behind it. Our detailed, point-by-point responses are provided below in blue. We truly appreciate your time, effort, and valuable input throughout the review process.

Response to reviewer 1:

The manuscript "Novel strains of tomato spotted wilt orthotospovirus (TSWV) are transmitted by western flower thrips in a context-specific manner" reports on the differences of thrips (Frankliniella occidentalis - WFT) transmission efficiencies among several resistance-breaking (RB) TSWV strains. This study follows a previous investigation reporting positive effects of the same RB strains on the WFT fitness. This new study provides evidence that WFT transmits some RB strains at a higher rate than non-RB strains, and WFT males showed to be more efficient vectors than females.

The authors addressed most of the issues raised in the first version of the manuscript. I keep my opinion that the inoculation efficiency analysed for different RB strains, male vs female thrips, and different IAPs should be firstly put in correlation with the acquisition efficiency: if the thrips differently acquired TSWV at the above-mentioned conditions this would influence the results of the inoculation efficiency experiments. I cannot see any consideration in Results, Discussion, and Figure 2 about the comparison of pre- and post-IAP results.

We thank Reviewer 1 for recognizing the novel aspects of our study and for acknowledging how prior comments were addressed. We agree that acquisition efficiency is a critical factor influencing inoculation outcomes. To minimize variability in virus acquisition, we used synchronized cohorts of thrips larvae (both male and female), all subjected to identical acquisition access periods (AAPs) on the same infected leaf tissue.

While acquisition efficiency was measured separately for each group and strain, our primary objective was to assess relative inoculation efficiency between sexes and IAP events within each strain under standardized acquisition conditions. Thus, any observed differences in

Texas A&M AgriLife Research
6500 W. Amarillo Blvd
Amarillo, TX 79106-1769
Tel. 806.354.5806
www.tx.ag/gadhavelab

inoculation efficiency across RB strains, sexes, or IAP events likely reflect post-acquisition dynamics rather than differences in acquisition itself.

For these reasons, we present the data from an "inoculation-centric" perspective, as it directly reflects the central step in transmission, while still accounting for acquisition in a controlled and robust manner.

INTRODUCTION:
Line 41: to the best ok my knowledge, "produce" is a verb not a noun. The authors should replace it with a noun, like product or production.

L41: The word "produce" is replaced with "product"

References 27-31: not all the references refer to F. occidentalis whereas the sentence in the text refers to this species only. This should be solved.

L64. Following this suggestion, the previous reference number 29, which was the only one referring to *F. fusca*, has been removed.

Line 78: "where viruliferous WFT feed on a series of leaf discs"

L78. The text is rephrased.

Line 103: "The non-RB strain, which is unable to overcome resistance conferred by Sw-5b or Tsw,"

L103-104. The text is rephrased as suggested.

Figures: you may use the letter annotation and indicate the level of significance into the legend, this would simplify the readability of the figures.

We sincerely appreciate the reviewer's suggestion. However, we believe the current figures effectively convey the significant differences between treatments and their levels in a clear and accessible manner. Including both letter annotations within the figures and significance levels in the legend may compromise overall readability and visual clarity. We have aimed to strike a balance between detail and interpretability and feel the current format best serves that purpose.

Line 382: the sentence: "Overall, when the females and males combined, RB strains inoculated with higher efficiency than the Non-RB stain." still lacks the meaning.

L382-383. The text has been revised for more clarity.

We sincerely thank Reviewer #3, Dr. Kishorekumar Reddy, for recommending our manuscript for publication.

We look forward to hearing from you.

Yours sincerely,

Kiran Gadhave, PhD, FRES
(Assistant Professor)

Texas A&M AgriLife Research
6500 W. Amarillo Blvd
Amarillo, TX 79106-1769
Tel. 806.354.5806
www.tx.ag/gadhavelab
